# TRANSDISCIPLINARY WORKING FOR CULTURE CHANGE IN ETHICAL AGETECH

**DOI:** 10.1093/geroni/igad104.1603

**Published:** 2023-12-21

**Authors:** Charlene Chu, Judith Sixsmith, Mei Lan Fang, Jennifer Boger

**Affiliations:** University of Toronto, Toronto, Ontario, Canada; University of Dundee, Dundee, Scotland, United Kingdom; University of Dundee, Dundee, Scotland, United Kingdom; University of Waterloo, Waterloo, Ontario, Canada

## Abstract

This presentation proposes a new way of understanding ethical culture in AgeTech as a dynamic and changing set of ethical research, design, and developmental processes and practices. By focusing on dynamics of ethical performance and the values, beliefs, and expectations that underpin them, this approach emphasizes ongoing ethical negotiation of all stakeholders involved in the research, design, and development of technology, rather than as a checklist at the research outset. Transdisciplinary working (TW) is proposed as an effective means to implement ethical processes and practices. By involving diverse stakeholders in the development of shared aims and objectives, ethical considerations become detached from the domain of researchers and become a more inclusive stakeholder negotiation. TW acknowledges that the development of new technologies cannot be separated from the people who design and use them; and the social practices, social norms, and social meanings in which they are steeped. The co-creation of socio-technical AgeTech systems is a key strategy for creating culture-change in ethical working environments. By involving diverse stakeholders in dynamic ethical processes across the research pathway, meaningful involvement of all stakeholders can be ensured, resulting in the effectiveness and relevance of AgeTech. Subsequently, technologies that are more responsive to the needs and desires of older people, carers, and professionals, and better reflect the complex ethical landscape in which they operate are created. Overall, this presentation offers a new perspective on ethical culture in AgeTech, one that involves diverse stakeholders in the creation of socio-technical systems that are both effective and ethical.

